# Measuring relatives’ perceptions of end-of-life communication with physicians in five countries: a psychometric analysis

**DOI:** 10.1007/s10433-022-00742-x

**Published:** 2022-11-13

**Authors:** Maciej Koniewski, Ilona Barańska, Violetta Kijowska, Jenny T. van der Steen, Anne B. Wichmann, Sheila Payne, Giovanni Gambassi, Nele Van Den Noortgate, Harriet Finne-Soveri, Tinne Smets, Lieve Van den Block, Katarzyna Szczerbińska, PACE project

**Affiliations:** 1 Institute of Sociology, Jagiellonian University, Kraków, Poland; 2 Laboratory for Research On Aging Society, Department of Sociology of Medicine, The Chair of Epidemiology and Preventive Medicine, Medical Faculty, Jagiellonian University Medical College, Skawińska 8 Street, 31-066 Kraków, Poland; 3 Department of Public Health and Primary Care, Leiden University Medical Center, Leiden, The Netherlands; 4 Department of Public and Occupational Health, Amsterdam Public Health Research Institute, Amsterdam UMC-VU University Medical Center (Department During the Study), Amsterdam, The Netherlands; 5 Department of Anesthesiology, Pain & Palliative Medicine, Radboud University Medical Center, Nijmegen, The Netherlands; 6 International Observatory On End of Life Care, Faculty of Health and Medicine, Lancaster University, Lancaster, UK; 7 Fondazione Policlinico Universitario A. Gemelli IRCCS, Rome, Italy; 8 Dipartimento Di Medicina E Chirurgia Traslazionale, Università Cattolica del Sacro Cuore, Rome, Italy; 9 Department of Geriatric Medicine, Ghent University Hospital, Ghent, Belgium; 10 Finnish Institute for Health and Welfare, Helsinki, Finland; 11 VUB-UGhent End-of-Life Care Research Group, Department of Family Medicine and Chronic Care, Vrije Universiteit Brussel (VUB), Brussels, Belgium

**Keywords:** Physician–patient relation, Family caregivers, Nursing home, Terminal care, Validation study, Cross-sectional Study

## Abstract

*The Family Perceptions of Physician-Family Caregiver Communication scale* (FPPFC) was developed to assess quality of physician-family end-of-life communication in nursing homes. However, its validity has been tested only in the USA and the Netherlands. The aim of this paper is to evaluate the FPPFC construct validity and its reliability, as well as the psychometric characteristics of the items comprising the scale. Data were collected in cross-sectional study in Belgium, Finland, Italy, the Netherlands and Poland. The factorial structure was tested in confirmatory factor analysis. Item parameters were obtained using an item response theory model. Participants were 737 relatives of nursing home residents who died up to 3 months prior to the study. In general, the FPPFC scale proved to be a unidimensional and reliable measure of the perceived quality of physician-family communication in nursing home settings in all five countries. Nevertheless, we found unsatisfactory fit to the data with a confirmatory model. An item that referred to advance care planning performed less well in Poland and Italy than in the Northern European countries. In the item analysis, we found that with no loss of reliability and with increased coherency of the item content across countries, the full 7-item version can be shortened to a 4-item version, which may be more appropriate for international studies. Therefore, we recommend use of the brief 4-item FPPFC version by nursing home managers and professionals as an evaluation tool, and by researchers for their studies as these four items confer the same meaning across countries.

**Supplementary Information:**

The online version contains supplementary material available at 10.1007/s10433-022-00742-x.

## Introduction

One of the key elements of end-of-life care is communication between physicians and patients’ families, especially when patients are unable to make decisions concerning the care they would like to receive (Biola et al. 2007; Gonella et al. 2019; Heyland et al. 2006). Yet, relatives of nursing home residents frequently report dissatisfaction with their communication with physicians (Shield et al. 2005).This calls for easy to use, valid and reliable physician-family communication evaluation tools, for managers and staff to assess families’ perceptions and expectations, and for researchers to better understand the role that physician-family communication plays in end-of-life care.

There are several scales measuring quality of physician-family communication in acute care settings, when patients are unable to speak for themselves (Cicekci et al. 2017). Also, there are numerous tools for assessing the quality of communication between a physician and a patient (Sustersic et al. 2018; Zill et al. 2014). However, the nature of the care provided in nursing homes, including end-of-life care, is different from care provided in hospitals or home. Residents are often frail or cognitively impaired, and goals of care (Zimmerman et al. 2015) may differ as well. Hence, specific tools are needed (van Soest-Poortvliet et al. 2012; Zimmerman et al. 2015) to assess communication between professionals and residents’ relatives in nursing homes.

The *Family Perceptions of Physician-Family Caregiver Communication scale* (FPPFC) has been designed to assess families’ perception of communication with a physician in nursing home settings (Biola et al. 2007; van Soest-Poortvliet et al. 2012; Zimmerman et al. 2015). It consists of seven items (Supplementary Table 1) assessed on a rating scale with 1 representing “strongly disagree,” 2 “disagree,” 3 “agree,” and 4 “strongly agree.” As all items are phrased in a positive manner, the higher the score, the better the perceived quality of communication. The FPPFC has been used in a few empirical studies to date (Barańska et al. 2020a, b; Biola et al. 2007; Boogaard et al. 2017; Cohen et al. 2012; Williams et al. 2008; Zimmerman et al. 2015). Some studies sought factors associated with the FPPFC score (Barańska et al. 2020b; Biola et al. 2007), other studies compared the quality of communication in different countries using the FPPFC (Barańska et al. 2020a; Cohen et al. 2012). High reliability was reported for the FPPFC, e.g., *α* = 0.93 in the Netherlands (van Soest-Poortvliet et al. 2012) and 0.89–0.96 in the USA (Biola et al. 2007; Zimmerman et al. 2015). Van Soest-Poortvliet et al. examined the criterion validity of the FPPFC (2012). Zimmerman et al. confirmed the one-factor FPPFC structure to fit the US data satisfactorily (Zimmerman et al. 2015). However, its validity has not been tested more broadly in other countries.

In the project: “Comparing the effectiveness of PAlliative Care for Elderly people in long-term care facilities in Europe” (PACE), we translated FPPFC scale, a version proposed by Zimermann et al. (2016), into five languages (Flemish, Dutch, Finnish, Italian and Polish) using the European Organization for Research and Treatment of Cancer guidelines (Dewolf et al. 2009; Van den Block et al. 2016 ). Translations are available in Supplementary Table 2. The aim of this paper is to evaluate the FPPFC construct validity and its reliability, as well as the psychometric characteristics of the items comprising the scale.

## Methods

### Study design, settings and participants

We used the after-death data from the PACE project (Van den Block et al. 2016). Data were collected in a questionnaire-based cross-sectional survey in 2015 in nursing homes in six countries: Belgium, England, Finland, Italy, the Netherlands and Poland. Stratified sampling of nursing homes was performed. In each participating nursing home, the residents who died in the last 3 months were identified. Then the questionnaire including FPPFC was sent to their relatives most involved in care. A returned questionnaire was considered as completed based on a valid informed consent. Due to a low number of answers from England (*n* = 25; 22% response rate), we limited the analyses to five countries. There were 273 nursing homes in these five countries, with 1539 residents reported to have died and 1341 of their relatives identified. The net response rate was 61%. We further excluded 77 respondents, as they answered less than 6 out of 7 items of the scale. Finally, we analyzed answers from 737 bereaved relatives in 222 nursing homes in five countries. The average age of the residents was 59 (SD = 11). Females comprised 64% of the sample. Almost two-thirds (64%) were the residents’ child, while 10% were the residents’ partner or spouse. Before admission, 22% of relatives had been living with the resident in the same house. Regarding education, 35% of the relatives completed primary or lower secondary education, 37% upper secondary or higher education, and 28% tertiary education. Detailed characteristics of the relatives, residents, and physicians in different countries are reported elsewhere (Barańska et al. 2020a).

To assess psychometric properties of the FPPFC scale, we applied a multi-step analytic process including calculating reliability coefficients, confirming unidimensionality, testing model fit to our multi-country data, testing alternative models (shorter scale’s versions) and using graphic methods to help decide which items could be dropped.

### Testing unidimensionality and reliability

The FPPFC has been considered a unidimensional measure (i.e., measuring a single construct). Among available methods to test unidimensionality, coefficient Cronbach’s alpha (*α*) (Cronbach 1951) is the most frequently used one (Dunn et al. 2014; Hattie 1985). It was reported in all studies which used the FPPFC (Barańska et al. 2020a, b; Biola et al. 2007; Boogaard et al. 2017; Cohen et al. 2012; van Soest-Poortvliet et al. 2012, 2013; Williams et al. 2008; Zimmerman et al. 2015). However, it is generally agreed that *α* is a poor index of unidimensionality (Revelle and Zinbarg 2009; Sijtsma 2009; Zinbarg et al. 2005; Cortina 1993). There are several, albeit infrequently utilized alternative indices. The coefficients beta (*β*) (Revelle 1979) and omega hierarchical (*ω*_h_) (McDonald 2013) are more appropriate than α, especially if the scale has any “microstructure.” The omega total (*ω*_t_) is a better estimate of the reliability than *α* (Revelle 2018).

Besides, *β* and *ω*_h_, the amount of explained common variance (ECV) (Bentler 2009; Ten Berge and Sočan 2004) of the general factor, is suggested as an index of the extent to which the scale measures one common construct (Sijtsma 2009). Since *α*, *β* and *ω*_h_ are formally nonequivalent (Zinbarg et al. 2005), important information about the psychometric properties of a scale may be missing when only *α* is reported. Therefore, in this article we reported *β*, *ω*_h_, *ω*_t_ and ECV, along with *α*. For all reported indices, the values range from 0 to 1, with 0 indicating no reliability in case of *ω*_t_ and *α*, whereas in case of ECV, *β*, *ω*_h_0 indicates no common factor presence and 1 reflecting perfect reliability and all variance explained by a general factor, i.e., no specific factors presence (Reise et al. 2010). As for reference values, essential unidimensionality could be claimed if the *β*, *ω*_h_, and ECV exceed 0.70–0.80 (Rodriguez et al. 2016a, b). The reliabilities should not be below .80, given the purpose of the FPPFC scale use (Carmines and Zeller 1979; Lance et al. 2006).

### Testing factorial structure

Additional to reporting unidimensionality and reliability indices of the FPPFC, we ran confirmatory factor analyses (CFA), which investigates whether the empirical data fits a specified theoretical model—in this case, the one-factor structure, suggested by Biola et al (Biola et al. 2007). Specifics of the analyses were described in the Supplementary File 2. Model fit was assessed with three commonly used fit indices (Muthen and Satorra 1995): root mean square error of approximation (RMSEA), comparative fit index (CFI), and the Tucker–Lewis index (TLI). As suggested by Hu and Bentler (Hu and Bentler 1999), we assumed that CFI and TLI values not lower than 0.95 (the closer to 1, the better), and RMSEA values not higher than 0.06 (the closer to 0, the better) indicate a good fit.

**Fig. 1:**
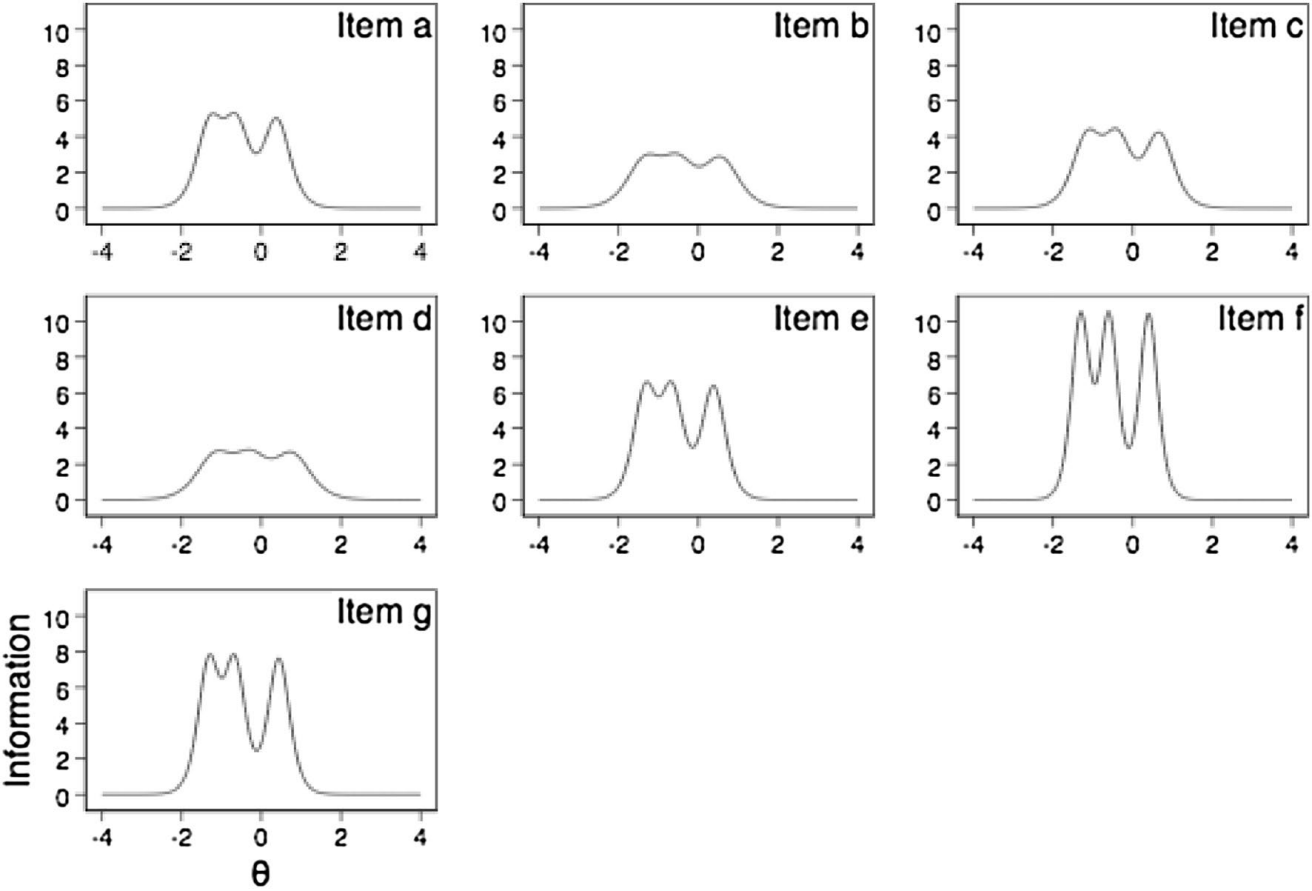
Item information functions of FPPFC items. The *y*-axis describes the information how much each item contributes to the measured construct, while *x*-axis (Theta) represents continuum of the communication quality perceived by respondents. Since we measure the respondents’ perception of communication quality, which is the latent variable, we impose an arbitrarily standardized scale with a mean of zero and the standard deviation as a unit. Abbreviations: FPPFC = Family Perception of Physician-Family Communication (range from 1 to 4). FPPFC items: see Table 2

We also used item response theory (IRT) models to assess discrimination parameters of items and to plot “item information functions”. For these purposes, we used a graded response model (Samejima 1997) for the total and countries’ samples. Despite serving different objectives and being based on different assumptions a single-factor CFA on ordinal items is formally equivalent to the graded response model (Samejima 1969). IRT is helpful to decide by which items to shorten the scale, and “item information function” graphs show which items are particularly useful (tall ones show higher discrimination power), and which are not (flat ones) (Fig. 1). More information about these methods is included in Supplementary File 2. We assessed the construct validity of the scale with use of CFA and IRT in the overall sample but also by country. Detailed results are presented in Table 2, Supplementary Table 4, and Supplementary Table 5.

## Results

Descriptive statistics, unidimensionality measures and reliability indices are provided in Table 1, and Supplementary Table 3 provides item frequencies. In all countries, except Finland (0.76), the ECV exceeded 0.80, which strongly suggests unidimensionality, as there is not much common variance beyond the general factor. The FPPFC general factor was a reliable measure of a single latent construct in Poland and the Netherlands, as its *ω*_h_ was greater than 0.90. Yet, in other countries (except Finland) it was close to 0.90. Coefficient *β*, which indicates the percentage of the scale that measures a single construct, had a value greater than 0.90 in all countries except Italy (0.66) and Finland (0.885).

**Table 1:** Descriptive statistics, unidimensionality and reliability indices of the 7-item FPPFC

	Belgium	Finland	Italy	Netherlands	Poland	Total
*n*	198	128	106	185	120	737
Mean	2.853	2.413	3.240	3.099	2.806	2.886
SD	0.850	0.837	0.685	0.706	0.819	0.827
*α*^a^	0.973 (0.956)	0.955 (0.938)	0.950 (0.909)	0.969 (0.949)	0.979 (0.963)	0.968 (0.951)
*ω*_h_	0.866	0.839	0.881	0.904	0.919	0.892
ω_t_	0.987	0.977	0.974	0.985	0.993	0.979
*β*	0.936	0.885	0.661	0.917	0.948	0.926
ECV	0.813	0.760	0.803	0.842	0.853	0.852

*FPPFC* Family Perception of Physician-Family Communication (range from 1 to 4); *SD* standard deviation; *ω*_*h*_ omega hierarchical; *ω*_*t*_ omega total; *β* coefficient beta; *ECV* explained common variance

^a^Coefficient *α* (in brackets not corrected for attenuation)

Despite unidimensionality being confirmed, a simple one-factor model, which comprises all 7 items, did not result in a satisfactory fit overall (χ^2^(14) = 788.346, RMSEA = 0.274, 90% CI [0.258, 0.290], CFI = 0.987, and TLI = 0.981). Similarly, country models did not fit our data well (Supplementary Table 4).

Guided by the results of the additional exploratory factor analysis and high values of modification indices, we tested different models. These included: extracting additional factors, allowing cross-loadings and error correlations, imposing a bi-factor structure. None of these resulted in considerable fit improvement. Therefore, we considered deleting some items. Natural candidates were items with the lowest discrimination parameter value (essentially a discrimination parameter in IRT is the same as factor loading in CFA), i.e., contributing little to the latent construct or, in other words, items of little help in distinguishing between those who evaluate quality of communication with physicians as good from those who evaluate as poor.

Table 2 reports item discrimination parameters obtained in the item response theory (IRT) models. Table 2 and Fig. 1 show that items b, c and d contributed the least to forming the general factor of physician-family communication relatively to the other items. Worth noting are differences between countries regarding which elements influenced the perceived communication quality the most. For example, whether a physician is a good listener or not (Table 2, item f), affected the physician-family communication perception the most in Belgium, the Netherlands and Poland. On the other hand, discussing resident’s end-of-life wishes was the least important in Italy.

**Table 2:** FPPFC item discrimination parameters estimated in IRT

Item^a^	Belgium	Finland	Italy	Netherlands	Poland	Total
a	4.253 (0.792)	3.974 (0.713)	5.415 (2.006)	3.874 (0.714)	4.524 (0.823)	4.323 (0.25)
b	3.909 (1.015)	2.372 (0.413)	5.486 (1.592)	3.358 (0.598)	5.223 (1.748)	3.26 (0.575)
c	3.995 (1.029)	2.899 (0.615)	6.026 (1.799)	3.574 (0.682)	5.406 (1.851)	3.973 (0.286)
d	3.724 (0.602)	2.615 (0.514)	1.219 (0.322)	3.8 (0.682)	5.911 (1.498)	3.135 (0.627)
e	5.112 (1.583)	3.644 (0.719)	4.2 (1.156)	6.439 (1.251)	3.914 (0.99)	4.868 (0.402)
f	6.02 (2.091)	4.938 (1.121)	3.543 (1.064)	7.61 (2.021)	7.727 (3.353)	6.201 (0.633)
g	5.424 (1.434)	5.067 (1.182)	3.269 (0.903)	5.551 (0.985)	4.954 (1.284)	5.31 (0.327)

Standard errors are reported in brackets

*FPPFC* Family Perception of Physician-Family Communication (range from 1 to 4); *IRT* item response theory

IRT, item response theory: graded response models run separately for each country and one model for all countries but with standard errors adjusted for clustering observations in countries

^a^FPPFC items: (a) The doctor always kept you or other family members informed about your relative’s condition. (b) You or other family members always received information from the doctor about what to expect while your relative was dying. (c) Your relative’s doctor always helped you or other family members to understand what he or she was saying to you about what to expect while your relative was dying. (d) The doctor always spoke to you, other family members or your relative about your relative’s wishes for medical treatment at the end of life. (e) You, other family members or your relative always had the opportunity to ask questions to the doctor about your relative’s care. (f) The doctor always listened to what you, other family members or your relative had to say about his/her medical treatment and end-of-life care. (g) The doctor always understood what you, other family members and your relative were going through

Based on these findings, we decided to test three models: first without items c and d, second without items b and c, third without items b, c, and d. Data fit of the first model was poor: χ^2^(5) = 92.368, RMSEA = 0.154, 90% CI [0.127, 0.182), CFI = 0.999, TLI = 0.997. It was slightly better for the second model though: χ^2^(5) = 65.612, RMSEA = 0.128, 90% CI [0.102, 0.157], CFI = 0.999, TLI = 0.998. The third model also had poor fit: χ^2^(2) = 29.832, RMSEA = 0.137, 90% CI [0.097, 0.183], CFI = 1, TLI = 0.999 (for country results see Supplementary Table 4). Although fit was not satisfactory, the three models of shortened scales fitted our data better than the full 7-item model. The lower 90% CI of RMSEA in the third model (Supplementary Table 4), for Finland dropped to 0.050 and for Belgium to 0.066, indicating acceptable (Finland) and almost acceptable fit (Belgium).The shortened scales had higher values of reliability coefficients *α* and *ω*_t_ comparable to the full 7-item version (Supplementary Table 5). The distributions and mean estimates of the scores were very similar for the full and shortened versions (Supplementary Figs. 1 & 2).

## Discussion

Based on high values of ECV, *ω*_h_ and β (indices of unidimensionality) and of *α* and *ω*_t_ (index of reliability) the FPPFC scale proved to be a unidimensional and reliable measure of the perceived quality of physician-family communication in nursing homes across Belgium, Finland, Italy, the Netherlands and Poland. However, reported values of unidimensionality and reliability indices might be inflated due to the local independence violation and left skewness of raw data. We are not familiar with any studies tackling the issue of ECV, *ω*_h_, *β*, *α* and *ω*_t_ robustness to the local independence violation and data skewness.

We found that the scale can be shortened without loss of its psychometric qualities with similar distributions and mean item scores. Shortened version does not include item responsible for local independence violation. A shortened 4-item version exhibited reliability indices values as high as the full 7-item version. We believe it might be recommended to be used:

1. by nursing home managers as an evaluation tool to identify areas where communication with a physician is scored lower. This is an easy and less expensive method allowing for quick collecting information from a large (or representative) group of respondents when conducting audit and looking for a feedback. The respondents may feel comfortable with anonymized answering to assess the quality of the communication with a physician;
2. by physicians for receiving feedback on their communication skills, and
3. by researchers, especially when the scale is administered as part of a long questionnaire. Use of the shorter scale may reduce risk of respondent fatigue and careless responding.

In some situations, open interviews with family members might be more appropriate to learn more in depth on their values and expectations concerning end-of-life communication. Both quantitative and qualitative methods may be helpful to study quality of end-of-life communication, although the last one is more expensive, time consuming, may generate some recruitment difficulties and ethical problems (Gonella et al. 2020).

However, shortening of the scale may raise concerns about whether theoretical construct will be maintained. The items b, c, d are the important parts of the concept of communication at the end of life. Items b and c refer to the increasing patients’ and their family members' awareness of the nature of the illness and impending death. Item d covers one of the goals of end-of-life communication, which is to create a shared understanding about patient’s values and treatment preferences and to plan care that is consistent with them. Studies on communication at the end-of-life demonstrate that family and patients want to be involved in the planning and decision-making process in the last stage of life (Sopcheck and Tappen 2021; Gjerberg et al. 2015). Therefore, these items had been included in the original FPPFC scale. However, family members’ perception of the communication with a physician relays on their expectations what they want to discuss with a professional. In some countries, family members do not expect discussing patient’s end-of-life preferences—there is still “a taboo”—and probably therefore they do not value communication with a physician by this context. Therefore, cultural context should be taken into account while assessing perceptions in different countries. Also, actions taken at the public level such as the promotion of the "Five Wishes Paper" raise awareness of the importance of communication at the end of life and may increase openness to talk about dying and thus generate expectations from patients and families on this issue.

Despite its unidimensionality, in the confirmatory model accounting for graded nature of the data we found unsatisfactory fit of the FPPFC full and shortened versions with our data. The fit indices were far from that as had been reported by Van Soest-Poortvliet al. (2012) on data from the US (RMSEA=0.06, NFI=0.89). Clearly, the scale has some microstructure, which make it a little unstable across countries when the measurement error is ruled out. Nevertheless, for two shortened versions (adefg-items and aefg-items) models fit the Finish and Belgian data better than the data from other countries. One possible reason might be that relatives’ expectations in regard communication with medical staff concerning close kin who is near to die may differ between countries due to cultural differences, as mentioned above.

The other explanation for the differences between countries may lie in the level of implementation of palliative care policies and services in nursing homes (Froggatt et al. 2017). Froggatt et al. (2017) showed that Poland, Finland or Italy are among the countries with a minimal level of palliative care activity within nursing homes as opposed to the Netherlands, Belgium or England. In some countries, there is a lack of adequate solutions encouraging end-of-life communication and advanced care legal directives. Differences in accessibility of palliative care in the nursing homes (ten Koppel et al. 2019) and in knowledge of palliative care principles among nursing home staff (Smets et al. 2018) may partially explain different extend of use of the end-of-life communication in these settings.

### Poor performance of the item regarding end-of-life wishes

Item d (referring to talk about resident’s end-of-life wishes) fits the construct underlying the FPPFC worst. It had the lowest value of the discrimination parameter in data from Belgium, Finland, and Italy. We argue that this may be due to the fact that item d could mean different things to respondents across countries, depending on how and to what extent the topic of end-of-life wishes is present in the public and private discourse, as well as the level of social awareness and related legal regulations. For example, some respondents may confound this question with issues that are not socially or legally accepted, e.g., euthanasia (Seymour et al. 2004) and thus provide answers in a socially desirable manner. The differences in answers to item d could also stem from the fact that, in some countries, physicians are used to talk about end-of-life wishes with residents or residents’ families (Andreasen et al. 2019), while in other countries where this topic is not normally explicitly discussed, relatives might not expect physicians to talk about it, and therefore they might not perceive this as a component of communication with a physician that affects its quality. This is supported by the fact that the discrimination parameters (hence “information power”) of item d in the model for the Netherlands, Belgium and Finland proved to be higher than in the model run on the subsample from Poland and Italy, i.e., 3.6–2.4, respectively. Written advance directives are more often obtained from nursing home residents in the Netherlands, Belgium and Finland compared with Poland and Italy (Andreasen et al. 2019). Hence, we suggest omitting the item regarding end-of-life wishes from the scale, especially when it is used to make comparisons between countries, taking into account cultural differences and public awareness, understanding and acceptance of the concept of advance care planning.

We acknowledge that determining wishes of persons nearing death, is one of the basic elements of palliative care. Therefore, we suggest that questions devoted to this topic should be adjusted to countries’ legal, cultural and health systems, e.g., more specific questions, narrowed down to various aspects of care: “do not hospitalize,” “request to try all life prolonging measures.”

### Possible further scale development

The full scale had a structural problem, i.e., the local independence assumption is violated for items b and c. Item b concerns receiving information from the physician, while item c concerns understanding it. The prerequisite to understand the information is to receive it first. Hence, we suggest dropping item c from the scale. Further, item b relates to the resident’s dying, while the other questions refer to a wider time window of end-of-life care. An exact time frame might be added (e.g., last month, week or at resident’s end of life).The change of the item structure that the respondent speaks for him—or herself only, not also for other family members might be taken to consideration to increase consistency of the item content.

Steinhauser et al. showed that physicians tend to focus on physical aspects of end-of-life care, while families highlight the need of psychological and emotional support (2000).The importance of a physician’s social-emotional skills, e.g., showing understanding and empathy, has been recognized in communication models, e.g., the Calgary-Cambridge model (Kurtz et al. 2003) and other models (Derksen et al. 2013). The FPPFC comprises only one item (g), which represents physician’s empathy. It might be that more items on this aspect need further development and testing for a better balance of person-oriented and information-oriented aspects of physician-family communication in the scale.

### Strengths and limitations of the study

This study contributes to the existing literature in several ways. First, it benefitted from stratified representative sampling, and the sample was considerably larger than those used in the previous studies (Cohen et al. 2012; van Soest-Poortvliet et al. 2012). Second, it provides multi-country comparisons and we identified relevant differences in the data from different countries. Third, to evaluate the FPPFC, we used several statistical analyses including better indices than the traditional one used in earlier evaluations of the properties of the FPPFC. Our strategy for the scale assessment was in accordance with standards of measurement theory and practice (Food and Drug Administration [FDA] 2009; Powers et al. 2017).

Albeit rigorous, this study also has limitations. In previous studies, relatives reported quality of communication during the “last months” or “last 4 months” of a nursing home resident’s life (Cohen et al. 2012; van Soest-Poortvliet et al. 2012, 2013; Zimmerman et al. 2015), while in our project we did not provide an exact, specific time frame. However, timing of the questionnaire administration was no later than 3 months after the resident’s death, which is commonly accepted in end-of-life research in regard to family evaluations (De Gendt et al. 2013; Pivodic et al. 2016; Vandervoort et al. 2013).

We encountered some difficulties with identifying residents’ relatives and low social acceptance to approach bereaved people for research purposes (in England, only 22% respondents answered). However, we have reached an overall sufficient response rate (61%) for mailed questionnaires. Moreover, the non-response analysis based on the characteristics of deceased residents for the relatives who did and did not respond showed no significant differences, except for resident’s sex and place of death.

The sufficient minimal sample size recommended for accurate parameters estimates in unidimensional GRM is 300–500; see Introduction in Jiang et al. (2016). However, for low number of items and for low-stake application a smaller sample seems sufficient. Single country models were run on samples between 100 and 200. In all single country, GRM models of the full 7-items scale version only three items have standards errors higher than one third of the discrimination parameter estimate: item f in Poland and Belgium, item c in Poland, and item a in Italy. Standard errors to discrimination parameter ratio in full sample (*n* = 737) were between 6 and 20%.

Summarizing, the original FPPFC scale was elaborated and tested in USA and the Netherlands, where both health care professionals and patients and their families use to be better prepared and more ready to be informed about approaching dying and end-of-life issues. In the frame of the PACE project, we have got opportunity to conduct validation of this scale in five European countries. Our analysis showed that FPPFC scale needs more research for testing its psychometric properties with special consideration of cultural context. Bearing in mind that attitude to dying and informing about dying may differ between countries, we suggest that it may impact patients’ and their families’ expectation concerning end-of-life communication with healthcare professionals, and therefore use of shorter version of FPPFC scale might be more appropriate for performing international comparisons.

## Conclusions

Given the high values of ECV, *ω*_h_ and *β* overall and in each of five studied countries, we conclude that the FPPFC measures a unidimensional construct. Nevertheless, we found unsatisfactory fit to the data with a confirmatory model. With no loss of reliability, with increased coherency of the item content across countries, and with no meaningful change to the score distribution and mean score estimates, the full 7-item version can be shortened to a 4-item version. We also suggest omitting a highly relevant item about resident’s wishes for *end-of-life* care, which could be measured separately or modified to better address cultural differences.

## Supplementary Information

Below is the link to the electronic supplementary material.

Supplementary file1 (DOC 351 KB)

Supplementary file2 (DOC 48 KB)

## Data Availability

The corresponding datasets of this study are available from the corresponding author on reasonable request.

## Abbreviations

CFA: Confirmatory factor analyses
CFI: Comparative fit index
ECV: Explained common variance
FPPFC: The Family Perceptions of Physician-Family Caregiver Communication scale
IRT: The item response theory
PACE project: PAlliative Care for Elderly people in long-term care facilities in Europe
RMSEA: Root mean square error of approximation
TLI: Tucker–Lewis index
*α*: Coefficient Cronbach’s alpha
*β*: Coefficients beta
*ω*_h_: Omega hierarchical
*ω*_t_: Omega total
