# Supplementary material for: Measuring relatives’ perceptions of end-of-life communication with physicians in five countries: a psychometric analysis
Source: Eur J Ageing. 2022 Nov 13;19(4):1561–70. doi: 10.1007/s10433-022-00742-x (PMC9729495; doi:10.1007/s10433-022-00742-x)
Supplement: Supplementary file 1 — Supplementary file1 (DOC 351 KB) [file 10433_2022_742_MOESM1_ESM.doc]

**Supplementary File 1**

**Supplementary Table 1** The FPPFC item wording in the original version from Biola et al. (2007)^a^ and the modified version from Zimmerman et al. (2015)^b^.

Respondents answer the question: *To what extent do you disagree or agree that …*

(all items scored as 0 = strongly disagree, 1 = disagree, 2 = agree, 3 = strongly agree).

| Item | Wording from Zimmerman et al. (2015) adopted in PACE study | Wording from Biola et al. (2007) |
| --- | --- | --- |
| a | The doctor always kept you or other family members informed about *[resident’s]* condition. | The doctor always kept you or other family caregivers informed about *[the LTC resident’s]* condition. |
| b | You or other family members always received information from the doctor about what to expect while *[resident]* was dying. | You or other family caregivers always received information from the doctor about what to expect while *[the LTC resident]* was dying. |
| c | *[Resident’s]* doctor always helped you or other family members to understand what he or she was saying to you about what to expect while *[resident]* was dying. | You or other family caregivers understood what [the LTC resident’s] doctor was saying to you about what to expect. |
| d | The doctor always spoke to you, other family members or *[resident]* about *[his/her]* wishes for medical treatment at the end of life. | The doctor always spoke to you, other family caregivers, or *[the LTC resident]* about *[his/her]* wishes for medical treatment at the end of life. |
| e | You, other family members or *[resident]* always had the opportunity to ask questions to the doctor about *[his/her]* care. | You, other family caregivers, or *[the LTC resident]* always had the opportunity to ask questions to the doctor about *[LTC resident’s]* care. |
| f | The doctor always listened to what you, other family member or *[resident]* had to say about *[his/her]* medical treatment and end-of-life care. | The doctor always listened to what you, other family caregivers, or *[the LTC resident]* had to say about *[his/her]* medical treatment and end-of-life care. |
| g | The doctor always understood what you, other family members and *[resident]* was going through. | The doctor always understood what you, other family caregivers, or *[the LTC resident]* was going through. |

Abbreviations: FPPFC=Family Perception of Physician-Family Communication (range from 1 to 4); LTC=long-term care; PACE study=Palliative Care for Older People.

^a^Biola H, Sloane PD, Williams CS, et al. Physician Communication with Family Caregivers of Long-Term Care Residents at the End of Life. J Am Geriatr Soc. 2007; 55(6): 846–856.

^b^Zimmerman S, Cohen LW, Washington T, Ward K, Giorgio P. Evaluating measures and instruments for quality improvement in assisted living. Ann Long-Term Care. 2016; 24(9): 15–24.

**Supplementary Table 2** The FPPFC English items translated into Flemish (Belgium), Finish, Italian, Dutch (The Netherlands) and Polish.

| ***English version of FPPFC*** | | | | |
| --- | --- | --- | --- | --- |
| To what extent do you agree with the following statements about communication?  *(for each line, please tick a single box in the column that applies)* | | | | |
|  | strongly disagree | disagree | agree | strongly  agree |
| a. The doctor always kept you or other family members informed about your relative’s condition. | [_] | [_] | [_] | [_] |
| b. You or other family members always received information from the doctor about what to expect while your relative was dying. | [_] | [_] | [_] | [_] |
| c. Your relative’s doctor always helped you or other family members to understand what he or she was saying to you about what to expect while your relative was dying. | [_] | [_] | [_] | [_] |
| d. The doctor always spoke to you, other family members or your relative about your relative’s wishes for medical treatment at the end of life. | [_] | [_] | [_] | [_] |
| e. You, other family members, or your relative always had the opportunity to ask questions to the doctor about your relative’s care. | [_] | [_] | [_] | [_] |
| f. The doctor always listened to what you, other family members, or your relative had to say about his/her medical treatment and end-of-life care. | [_] | [_] | [_] | [_] |
| g. The doctor always understood what you, other family members and your relative were going through. | [_] | [_] | [_] | [_] |

| ***Flemish version (Belgium) of FPPFC*** | | | | |
| --- | --- | --- | --- | --- |
| In welke mate bent u het eens met de volgende uitspraken over communicatie?  *(per regel graag één vakje aankruisen in de kolom die van toepassing is)* | | | | |
|  | helemaal **niet** mee eens | niet mee eens | mee  eens | helemaal  **mee eens** |
| a. de arts hield u of andere familieleden altijd op de hoogte van de toestand van uw naaste | [_] | [_] | [_] | [_] |
| b. u of andere familieleden ontvingen altijd informatie van de arts over wat te verwachten was tijdens het sterven van uw naaste | [_] | [_] | [_] | [_] |
| c. de arts hielp u of andere familieleden te begrijpen wat hij of zij vertelde over wat te verwachten was tijdens het sterven | [_] | [_] | [_] | [_] |
| d. de arts sprak altijd met u, andere familieleden of uw naaste zelf over de wensen van uw naaste voor medische behandeling bij het einde van het leven | [_] | [_] | [_] | [_] |
| e. u, andere familieleden of uw naaste zelf hadden altijd de gelegenheid om vragen te stellen aan de arts over de verzorging van uw naaste | [_] | [_] | [_] | [_] |
| f. de arts luisterde altijd naar wat u, andere familieleden of uw naaste zelf te zeggen hadden over medische behandelingen en zorg in de laatste levensfase van uw naaste | [_] | [_] | [_] | [_] |
| g. de arts begreep altijd wat u, andere familieleden of uw naaste zelf doormaakten | [_] | [_] | [_] | [_] |

| ***Finnish version of FPPFC*** | | | | |
| --- | --- | --- | --- | --- |
| Missä määrin olette samaa mieltä seuraavien vuorovaikutusta koskevien väittämien kanssa?  (*Valitkaa ja rastittakaa kultakin riviltä mielestäsi parhaiten paikkansa pitävä vaihtoehto*) | | | | |
|  | täysin eri mieltä | eri mieltä | samaa mieltä | täysin samaa mieltä |
| a. Te tai muut kuolevaa omaistanne hoitaneet perheenjäsenet ja läheiset saitte aina ajantasaista tietoa lääkäriltä hänen voinnistaan | [_] | [_] | [_] | [_] |
| b. Te tai muut kuolevaa hoitaneet perheenjäsenet ja läheiset ymmärsitte, kun hoitava lääkäri kertoi mitä olisi odotettavissa | [_] | [_] | [_] | [_] |
| c. Edesmenneen läheisenne lääkäri auttoi aina teitä tai muita hänen hoitoonsa osallistuneista omaisia ymmärtämään, mitä edesmennyt läheisenne tarkoitti puhuessaan Teille siitä. mitä olisi odotettavissa hänen kuollessaan | [_] | [_] | [_] | [_] |
| d. Lääkäri piti kaikki teidät kuolevaa hoitaneet perheenjäsenet ja läheiset ajan tasalla siitä, miten kuoleva halusi itseään lääketieteellisesti hoidettavan elämän loppuvaiheessa | [_] | [_] | [_] | [_] |
| e. Kaikilla teillä kuolevaa hoitaneilla perheenjäsenillä ja läheisillä oli aina mahdollisuus kysyä lääkäriltä läheisenne hoidosta | [_] | [_] | [_] | [_] |
| f. Lääkäri kuunteli aina mitä kaikilla teillä kuolevaa hoitaneilla perheenjäsenillä tai läheisillä oli sanottavana hänen lääketieteellisestä hoidostaan ja loppuelämän hoivastaan. | [_] | [_] | [_] | [_] |
| g. Lääkäri ymmärsi aina, mitä kaikki te kuolevaa hoitaneet perheenjäsenet ja läheiset jouduitte kestämään | [_] | [_] | [_] | [_] |

| ***Italian version of FPPFC*** | | | | |
| --- | --- | --- | --- | --- |
| In che misura è d’accordo con le seguenti dichiarazioni in merito alla comunicazione?  *(barrare una casella per ogni riga nella colonna corrispondente))* | | | | |
|  | Fortemente contrario | In disaccordo | D'accordo | Assolutamente  d'accordo |
| a. Sia lei che gli altri membri della famiglia sono sempre stati informati sulle condizioni del paziente | [_] | [_] | [_] | [_] |
| b. Sia lei che gli altri membri della famiglia hanno sempre saputo cosa aspettarsi nella fase precedente al decesso del paziente | [_] | [_] | [_] | [_] |
| c. Il medico ha sempre aiutato sia lei che gli altri membri della famiglia a comprendere le questioni riguardanti cosa aspettarsi nella fase precedente al decesso del paziente | [_] | [_] | [_] | [_] |
| d. Il medico ha sempre riferito a lei, agli altri membri della famiglia e al paziente le volontà del suo parente in merito ai trattamenti medici da adottare nell'ultima fase di vita | [_] | [_] | [_] | [_] |
| e. È stato sempre possibile per lei, gli altri membri della famiglia e il suo parente porre domande al medico in merito alle cure mediche fornite al paziente | [_] | [_] | [_] | [_] |
| f. Il medico ha sempre ascoltato ciò che lei, gli altri membri della famiglia e il suo parente avevano da dire in merito ai trattamenti medici e alle cure della fase terminale | [_] | [_] | [_] | [_] |
| g. Il medico si è sempre mostrato comprensivo della situazione che lei, gli altri membri della famiglia e il suo parente stavano vivendo | [_] | [_] | [_] | [_] |

| ***Dutch version (the Netherlands) of FPPFC*** | | | | |
| --- | --- | --- | --- | --- |
| To what extent do you agree with the following statements about communication?  *(for each line, please tick a single box in the column that applies)* | | | | |
|  | helemaal  **oneens** | oneens | eens | helemaal  **eens** |
| a. de arts hield u of andere familieleden altijd op de hoogte van de toestand van uw naaste | [_] | [_] | [_] | [_] |
| b. u of andere familieleden ontvingen altijd informatie van de arts over wat te verwachten was tijdens het sterven van uw naaste | [_] | [_] | [_] | [_] |
| c. de arts hielp u of andere familieleden te begrijpen wat hij/zij vertelde over wat te verwachten was tijdens het sterven | [_] | [_] | [_] | [_] |
| d. de arts sprak altijd met u, andere familieleden of uw naaste zelf over zijn/haar wensen voor medische behandeling bij het einde van het leven | [_] | [_] | [_] | [_] |
| e. u, andere familieleden of uw naaste zelf hadden altijd de gelegenheid om vragen te stellen aan de arts over zijn/haar zorg | [_] | [_] | [_] | [_] |
| de arts luisterde altijd naar wat u, andere familieleden of uw naaste te zeggen hadden over zijn/haar medische behandelingen en levenseindezorg | [_] | [_] | [_] | [_] |
| f. de arts begreep altijd wat u, andere familieleden of uw naaste zelf doormaakten | [_] | [_] | [_] | [_] |

| ***Polish version of FPPFC*** | | | | |
| --- | --- | --- | --- | --- |
| W jakim stopniu zgadza się Pan(i) z następującymi stwierdzeniami dotyczącymi komunikacji?  *(w każdym wierszu proszę zaznaczyć jedno pole w odpowiedniej kolumnie)* | | | | |
|  | Zdecydowanie się nie zgadzam | Nie zgadzam się | Zgadzam się | Zdecydowanie się zgadzam |
| a. Lekarz zawsze informował mnie lub inne osoby z rodziny sprawujące opiekę na temat stanu zdrowia Krewnego. | [_] | [_] | [_] | [_] |
| b. Ja lub inni krewni zawsze otrzymywaliśmy informacje od lekarza, czego można się spodziewać, kiedy umiera bliska osoba. | [_] | [_] | [_] | [_] |
| c. Lekarz zawsze starał się, aby informacje, które przekazywał na temat tego, czego można się spodziewać, kiedy umiera bliska osoba, były dla nas zrozumiałe. | [_] | [_] | [_] | [_] |
| d. Lekarz zawsze rozmawiał ze mną, innymi osobami z rodziny lub moim Krewnym na temat jego życzeń w zakresie opieki medycznej pod koniec życia. | [_] | [_] | [_] | [_] |
| e. Ja, inne osoby z rodziny lub mój Krewny zawsze mieliśmy możliwość zadania lekarzowi pytań dotyczących opieki. | [_] | [_] | [_] | [_] |
| f. Lekarz zawsze słuchał, co ja, inne osoby z rodziny lub mój Krewny mieliśmy do powiedzenia na temat opieki medycznej pod koniec życia. | [_] | [_] | [_] | [_] |
| g. Lekarz zawsze rozumiał, przez co przechodzimy: ja, inne osoby z rodziny i Krewny. | [_] | [_] | [_] | [_] |

**Supplementary Table 3** Distribution of answers on FPPFC items.

| Item | n | 1  strongly disagree | 2  disagree | 3  agree | 4  strongly agree |
| --- | --- | --- | --- | --- | --- |
| a | 736 | 10.2% | 15.2% | 41.2% | 33.4% |
| b | 733 | 10.6% | 19.8% | 41.1% | 28.5% |
| c | 725 | 13.4% | 22.2% | 39.9% | 24.6% |
| d | 725 | 14.8% | 27.5% | 35.6% | 22.2% |
| e | 736 | 9.1% | 14.8% | 43.2% | 32.9% |
| f | 737 | 9.1% | 16.7% | 42.7% | 31.5% |
| g | 733 | 9.1% | 14.7% | 45.6% | 30.6% |

Abbreviations: FPPFC=Family Perception of Physician-Family Communication (range from 1 to 4).

**Supplementary Table 4** Fit statistics of tested FPPFC one-factor models.

|  | RMSEA | 90% CI | CFI | TLI |
| --- | --- | --- | --- | --- |
| **7-item model** |  |  |  |  |
| Belgium | .239 | .208–.272 | .989 | .983 |
| Finland | .218 | .179–.260 | .981 | .972 |
| Italy | .257 | .214–.303 | .983 | .975 |
| Netherlands | .261 | .228–.294 | .983 | .974 |
| Poland | .213 | .172–.256 | .993 | .989 |
| **5-item (a b _ _ e f g) model** |  |  |  |  |
| Belgium^a^ | .255 | .208–.305 | .995 | .991 |
| Finland | .152 | .085–.225 | .996 | .991 |
| Italy | .330 | .260–.406 | .988 | .976 |
| Netherlands | .163 | .110–.222 | .997 | .994 |
| Poland^a^ | .185 | .124–.253 | .996 | .994 |
| **5-item (a _ _ d e f g) model** |  |  |  |  |
| Belgium | .124 | .071–.183 | .999 | .998 |
| Finland | .114 | .040–.190 | .998 | .995 |
| Italy | .223 | .152–.300 | .994 | .987 |
| Netherlands | .240 | .187–.297 | .993 | .987 |
| Poland | .191 | .124–.265 | .997 | .995 |
| **4-item (a _ _ _ e f g) model** |  |  |  |  |
| Belgium^a^ | .066 | .000-.150 | 1 | 1 |
| Finland | .050 | .000-.190 | 1 | .999 |
| Italy | .393 | .285–.513 | .993 | .978 |
| Netherlands | .170 | .089–.264 | .999 | .996 |
| Poland^a^ | .228 | .144–.322 | .997 | .994 |

Abbreviations: FPPFC=Family Perception of Physician-Family Communication (range from 1 to 4); RMSEA=root mean square error of approximation; 90% CI=90% Confidence Interval; CFI=comparative fit index; TLI=Tucker–Lewis index.

^a^The model required modification to be estimable. The f-item standardized loading was fixed to 1 to attain a positive definite residual covariance matrix.

**Supplementary Table 5** Descriptive statistics and reliability indices of shortened FPPFC scale versions.

|  | Belgium | Finland | Italy | Netherlands | Poland | Total |
| --- | --- | --- | --- | --- | --- | --- |
| **5-items (a b _ _ e f g)** | | | | | | |
| n | 198 | 128 | 106 | 185 | 120 | 737 |
| Mean | 2.931 | 2.518 | 3.317 | 3.145 | 2.861 | 2.957 |
| SD | 0.857 | 0.877 | 0.696 | 0.708 | 0.809 | 0.832 |
| α^a^ | .965  (.941) | .947 (.927) | .955 (.920) | .961 (.933) | .968 (.946) | .960  (.939) |
| ω_t_ | .984 | .968 | .981 | .974 | .987 | .973 |
| **5-items (a _ _ d e f g)** | | | | | | |
| n | 198 | 128 | 106 | 185 | 120 | 737 |
| Mean | 2.907 | 2.403 | 3.237 | 3.141 | 2.836 | 2.914 |
| SD | 0.856 | 0.859 | 0.679 | 0.716 | 0.814 | 0.836 |
| α | .967  (.944) | .947  (.925) | .917  (.852) | .965  (.938) | .973  (.951) | .959  (.937) |
| ω_t_ | .976 | .965 | .949 | .980 | .991 | .973 |
| **4-items (a _ _ _ e f g)** |  |  |  |  |  |  |
| n | 198 | 128 | 106 | 185 | 120 | 737 |
| Mean | 2.982 | 2.461 | 3.334 | 3.178 | 2.899 | 2.978 |
| SD | 0.871 | 0.894 | 0.703 | 0.721 | 0.816 | 0.871 |
| α | .966  (.941) | .954  (.932) | .948  (.906) | .968  (.936) | .967  (.940) | .964  (.941) |
| ω_t_ | .975 | .969 | .971 | .981 | .988 | .978 |

Abbreviations: FPPFC=Family Perception of Physician-Family Communication (range from 1 to 4); SD=standard deviation; ω_t_=omega total.

^a^Coefficient α (in brackets not corrected for attenuation).


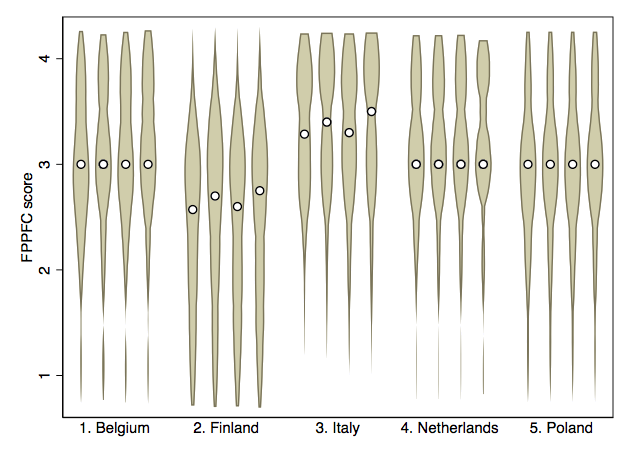


**Supplementary Fig. 1.** Distributions of the FPPFC scores calculated as arithmetical means with marked distribution medians. In each country panel distributions are, from left to right, for: full 7-item version, 5-item (abefg) version, 5-item (adefg) version, 4-item (aefg) version.

Abbreviations: FPPFC=Family Perception of Physician-Family Communication (range from 1 to 4).


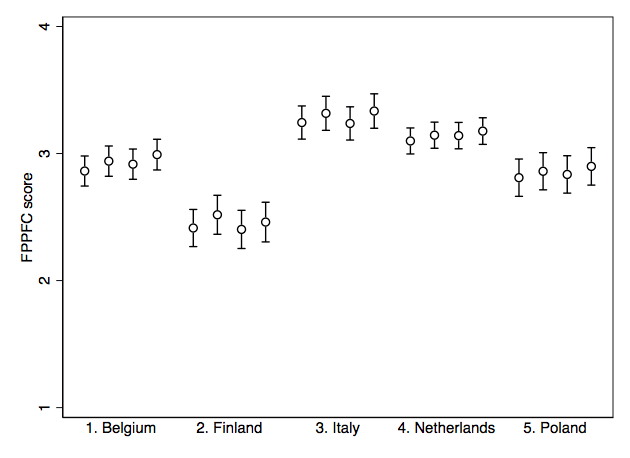


**Supplementary Fig. 2.** Mean score estimates of the FPPFC with their 95% confidence intervals. In each country panels represent, from left to right: full 7-item version, 5-item (abefg) version, 5-item (adefg) version, 4-item (aefg) version.

Abbreviations: FPPFC=Family Perception of Physician-Family Communication (range from 1 to 4).
