# Supplementary material for: Measuring relatives’ perceptions of end-of-life communication with physicians in five countries: a psychometric analysis
Source: Eur J Ageing. 2022 Nov 13;19(4):1561–70. doi: 10.1007/s10433-022-00742-x (PMC9729495; doi:10.1007/s10433-022-00742-x)
Supplement: Supplementary file 2 — Supplementary file2 (DOC 48 KB) [file 10433_2022_742_MOESM2_ESM.doc]

**Supplementary File 2**

**Additional information about the methods used**

For computing unidimensionality and reliability indices of the FPPFC we used commands “omega” and “iclust” available in “psych” package for R (Revelle 2018). All above-mentioned indices were estimated using polychoric correlation matrix, which is adequate for ordinal data. Estimates were bootstrapped with 1,000 replications.

The confirmatory factor analyses (CFA) were performed in Mplus 8.1 using the robust weighted least square (WLSMV) estimator, recommended for ordered categorical data (Flora and Curran 2004). The models for a joint sample of five countries accounted for the dependence of observations clustered within countries by adjusting the standard errors using a sandwich estimator (Muthen and Satorra 1995; Muthén and Muthén 2012). The sandwich estimator takes into account complex sampling features (e.g., clustering) when providing estimates of standard errors and χ^2^ tests of model fit. We used the sandwich estimator rather than a two-level model (i.e., observations nested within countries), because goodness of fit indices in two-level settings are likely to be dominated by the model fit at the lower level, and may not be sensitive to detect lack of fit at the higher level (Ryu 2014).

For estimating item discrimination parameters we used a graded response model (Samejima 1997) with logit link using robust marginal maximum likelihood estimator (i.e., MLR in Mplus) and country-cluster sandwich estimator for the joint model for all 5 countries. To plot “item information functions” of FPPFC items we used a graded response model with country-cluster sandwich estimator. We chose graded response model over generalized partial credit model, an alternative for graded data, based on lower BIC and AIC values. We used Stata MP 16.1 built-in procedures available under “irt” command.

**Local Item Independence**

Measurement theory requires items of a scale to be locally independent. It means that while controlling for the latent construct (in our case the “physician-family communication quality”), a response to a question is independent of responses to other items in a scale. However, as indicated by Zimmerman et al., if a family did not “always” receive information from the physician about what to expect while the resident was dying (per item b), then item c (i.e., physician always helped to understand what to expect while the resident was dying) should be coded “1”, i.e. “strongly disagree” (Zimmerman, Cohen, Washington, Ward and Giorgio 2016). Following this suggestion, and also based on the parallel phrasing of “always” in these two items, we recoded other answers provided on item c to “1” for 7 respondents who strongly disagreed with item b. This was necessary to maintain logical consistency of the data collected in self-administered questionnaires. However, this recoding did not solve the problem with local dependency between items b and c. We dealt with it by examining properties of a shortened scale version without item c.

Flora DB, Curran PJ (2004) An Empirical Evaluation of Alternative Methods of Estimation for Confirmatory Factor Analysis With Ordinal Data. Psychological Methods 9(4): 466–491. https://doi.org/10.1037/1082-989X.9.4.466

Muthen BO, Satorra A (1995) Complex Sample Data in Structural Equation Modeling. Sociological Methodology 25. https://www.statmodel.com/download/SMMuthenSatorra1995.pdf. Accessed 20 August 2019.

Muthén LK, Muthén BO (2012) Statistical Analysis With Latent Variables. Mplus user’s guide (7th ed.). Los Angeles. www.StatModel.com. Accessed 20 August 2019.

Revelle WR (2018) psych: Procedures for Personality and Psychological Research.

Ryu E (2014) Model fit evaluation in multilevel structural equation models. Frontiers in Psychology 5. doi:10.3389/fpsyg.2014.00081

Samejima F (1997) Graded Response Model. In: van der Linden WJ, Hambleton RK (ed) Handbook of Modern Item Response Theory. Springer, New York, pp 85-100.

Zimmerman S, Cohen LW, Washington T, Ward K, Giorgio P (2016) Evaluating measures and instruments for quality improvement in assisted living. Annals of Long-Term Care 24(9): 15–24.
